# Regulation (EU) 2017/746 (IVDR): practical implementation of annex I in pathology

**DOI:** 10.1007/s00292-023-01274-6

**Published:** 2023-12-04

**Authors:** Andy Kahles, Hannah Goldschmid, Anna-Lena Volckmar, Carolin Ploeger, Daniel Kazdal, Roland Penzel, Jan Budczies, Christa Flechtenmacher, Ulrich M. Gassner, Monika Brüggemann, Michael Vogeser, Peter Schirmacher, Albrecht Stenzinger

**Affiliations:** 1 Institute of Pathology, University Hospital Heidelberg, Im Neuenheimer Feld 224, 69120 Heidelberg, Germany; 2 Faculty of Law, University of Augsburg, Augsburg, Germany; 3 2nd Internal Medicine Department, Hematology Lab Kiel, University Hospital Schleswig-Holstein (UKSH), Kiel, Germany; 4 Institute of Laboratory Medicine, University Hospital, LMU Munich, Munich, Germany

**Keywords:** Quality Management, Quality Assurance in health care, Regulatory requirements, In-house manufacturing, Laboratory-developed tests, Qualitätsmanagement, Qualitätssicherung in der Gesundheitsversorgung, Regulatorische Anforderungen, Eigenherstellung, Laboratory-developed-Test

## Abstract

**Background:**

Regulation (EU) 2017/746 on in vitro diagnostic medical devices (IVDR) imposes several conditions on pathology departments that develop and use in-house in vitro diagnostic medical devices (IH-IVDs). However, not all of these conditions need to be implemented immediately after the IVDR entered into force on 26 May 2022. Based on an amending regulation of the European Parliament and the Council of the European Union, the requirements for IH-IVDs will be phased in. Conformity with the essential safety and performance requirements of annex I must be ensured from May 2022.

**Objectives:**

With this article, we would like to present the practical implementation of the currently valid conditions for IH-IVDs at the Institute of Pathology at the University Hospital of Heidelberg, in order to provide possible assistance to other institutions.

**Conclusions:**

In addition to the intensive work on the requirements for IH-IVDs, several guidance documents and handouts provide orientation for the implementation and harmonisation of the requirements for healthcare institutions mentioned in Article 5 (5). Exchange in academic network structures is also of great importance for the interpretation and practical implementation of the IVDR. For university and nonuniversity institutions, ensuring conformity with the IVDR represents a further challenge in terms of personnel and time, in addition to the essential tasks of patient care, teaching and research and the further development of methods for optimal and targeted diagnostics, as well as the maintenance of the constantly evolving quality management system.

## Abstract (de)

**Hintergrund:**

Die Verordnung (EU) 2017/746 über *In-vitro-*Diagnostika (IVDR) stellt mehrere Bedingungen an Pathologische Institute, die hausinterne *In-vitro-*Diagnostika (IH-IVD) entwickeln und anwenden. Diese Bedingungen müssen jedoch nicht alle unmittelbar mit dem Geltungsbeginn der IVDR zum 26.05.2022 umgesetzt worden sein. Auf der Grundlage einer Änderungsverordnung des Europäischen Parlaments und des Rates der Europäischen Union werden die Anforderungen an IH-IVD stufenweise eingeführt. Die Konformität mit den grundlegenden Sicherheits- und Leistungsanforderungen gemäß Anhang I muss seit Mai 2022 gewährleistet sein.

**Ziel der Arbeit:**

Mit diesem Artikel möchten wir die praktische Umsetzung der aktuell gültigen Bedingungen für IH-IVD im Pathologischen Institut des Universitätsklinikums Heidelberg vorstellen und damit mögliche Hilfestellung für andere Einrichtungen geben.

**Schlussfolgerungen:**

Neben der intensiven Auseinandersetzung mit den Anforderungen an IH-IVD geben mehrere Handreichungen und Hilfestellungen eine Orientierungshilfe zur Umsetzung und Harmonisierung der in Artikel 5 (5) genannten Anforderungen an Gesundheitseinrichtungen. Auch der Austausch in akademischen Netzwerkstrukturen ist für die Interpretation und die praktische Umsetzung der IVDR von großer Bedeutung. Für universitäre und nicht-universitäre Einrichtungen stellt die Sicherstellung der IVDR-Konformität – neben den wesentlichen Aufgaben in der Krankenversorgung, in der Lehre und der Forschung und Weiterentwicklung von Methoden zur optimalen und zielgerichteten Diagnostik sowie der Aufrechterhaltung des sich stetig weiterentwickelnden Qualitätsmanagementsystems  – eine weitere personelle und zeitliche Herausforderung dar.

## Introduction

“Regulation (EU) 2017/746 of the European Parliament and of the Council of 5 April 2017 on in vitro diagnostic medical devices and repealing Directive 98/79/EC and Commission Decision 2010/227/EU (Text with EEA relevance.)” [15] (short: IVDR) entered into force on 26 May 2017 and applied from 26 May 2022. The main objective of the regulation, which applies throughout the EU, is to ensure the highest possible level of patient health protection combined with a high level of user safety through harmonized requirements for the manufacture and use of in vitro diagnostic medical devices (IVD).

This article describes the procedure for implementing the currently applicable requirements of the IVDR for health institutions and the “General Safety and Performance Requirements” (IVDR, Annex I) into the established quality management (QM) system of the Institute of Pathology at the University Hospital of Heidelberg (IPH).

## Regulation 2027/746 (IVDR): implication for pathology departments

The IVDR distinguishes between two types of IVDR-compliant in vitro diagnostic medical devices (IVD; Table 1): CE-marked IVDs (CE-IVDs) from economic operators and in-house IVDs (IH-IVDs) from health institutions (see Table 2 for definitions of terms). For complex diagnostics in pathology, both types of IVDs, also in combination, are used to ensure optimal patient care.

**Table 1:** Regulation 2017/746 (IVDR) distinguishes between two types of in vitro diagnostic medical devices (IVDs) that are IVDR-compliant: CE-marked IVDs (CE-IVD) from economic operators and in-house IVDs (IH-IVDs) from health institutions

CE-IVDs	IH-IVDs
*Manufacturer: *economic operators *Placing: *European market	*Manufacturer: *health institutions (e.g., pathology departments) *Placing: *within the manufacturing health institution
*All *requirements of the IVDR must be met, depending on the type and risk class of the device	*Article 5 (5) *is to be fulfilled, including Annex I
“Notified bodies” are responsible for conformity assessment with the IVDR: Currently 10 × in the European Union (as of 05/2023 [6])	Without participation of notified bodies Monitoring by competent authority
Registration of the devices in the European Database on Medical Devices (EUDAMED) [5]	No EUDAMED registration

**Table 2:** Definitions according to Regulation 2017/746 (IVDR) [15] (in alphabetical order)

Term	Definition according to Regulation 2017/746 (IVDR) [15]	Reference
Analytical performance	‘analytical performance’ means the ability of a device to correctly detect or measure a particular analyte	Article 2 (40)
Benefit–risk determination	‘benefit–risk determination’ means the analysis of all assessments of benefit and risk of possible relevance for the use of the device for the intended purpose, when used in accordance with the intended purpose given by the manufacturer	Article 2 (17)
Clinical performance	‘clinical performance’ means the ability of a device to yield results that are correlated with a particular clinical condition or a physiological or pathological process or state in accordance with the target population and intended user	Article 2 (41)
Economic operator	‘economic operator’ means a manufacturer, an authorised representative, an importer or a distributor	Article 2 (28)
Health institution	‘health institution’ means an organisation the primary purpose of which is the care or treatment of patients or the promotion of public health	Article 2 (29)
Instructions for use	‘instructions for use’ means the information provided by the manufacturer to inform the user of a device’s intended purpose and proper use and of any precautions to be taken	Article 2 (14)
Intended purpose	‘intended purpose’ means the use for which a device is intended according to the data supplied by the manufacturer on the label, in the instructions for use or in promotional or sales materials or statements or as specified by the manufacturer in the performance evaluation	Article 2 (12)
Label	‘label’ means the written, printed or graphic information appearing either on the device itself, or on the packaging of each unit or on the packaging of multiple devices	Article 2 (13)
Manufacturer	‘manufacturer’ means a natural or legal person who manufactures or fully refurbishes a device or has a device designed, manufactured or fully refurbished, and markets that device under its name or trademark	Article 2 (23)
Performance evaluation	‘performance evaluation’ means an assessment and analysis of data to establish or verify the scientific validity, the analytical and, where applicable, the clinical performance of a device	Article 2 (44)
Performance of a device	‘performance of a device’ means the ability of a device to achieve its intended purpose as claimed by the manufacturer. It consists of the analytical and, where applicable, the clinical performance supporting that intended purpose	Article 2 (39)
Risk	‘risk’ means the combination of the probability of occurrence of harm and the severity of that harm	Article 2 (16)
User	‘user’ means any healthcare professional or lay person who uses a device	Article 2 (30)

Recital 29 of the IVDR describes the special importance of health institutions and the IVDs they develop themselves. According to this, health institutions—and thus pathology departments—should continue to have the possibility to manufacture, modify and use devices in-house, in order to be able to respond to the specific needs of the patient target groups. For this purpose, however, the IVDR is to prescribe EU-wide harmonized rules (Recital 28), which are described in Article 5 (5).

**Fig. 1:**
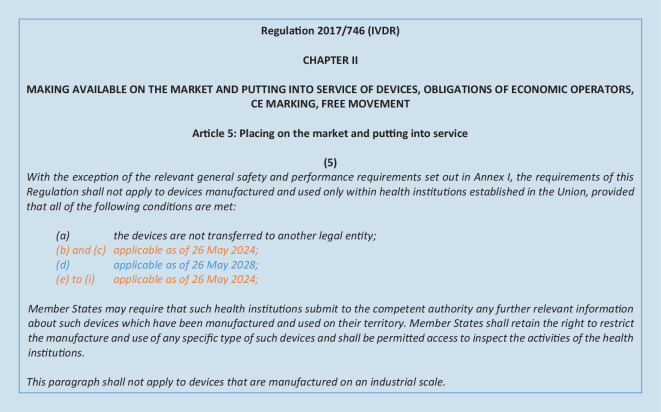
Currently applicable conditions for in-house in vitro diagnostic medical devices (IH-IVD) according to Regulation 2017/746 (IVDR), Article 5 (5). For conditions (b)–(i), a later start date was decided [16]

This article sets out several conditions for health institutions developing and using IH-IVDs. If all of the conditions described herein are met, any additional requirements of the IVDR do not apply to IH-IVDs. These conditions for health institutions are coming into effect gradually, following an amending regulation in January 2022 ([16]; Fig. 1).

The requirements summarized below must already be followed since 26 May 2022:

1. IH-IVDs must comply with the general safety and performance requirements (IVDR, Annex I).
2. The manufacture and use must take place within the EU.
3. IH-IVDs may only be used by the institution itself and may not be transferred to another legal entity.
4. Competent authorities shall be provided with relevant information on the devices upon request and shall have access to the health institutions to verify their activities.
5. IH-IVDs shall not be manufactured on an industrial scale.

The term “industrial scale” is not defined in the IVDR. However, the guideline document MDCG-2023‑1, which was prepared specifically for IH-IVDs by the Medical Device Coordination Group (MDCG) and published in January 2023, comments on this [13]. This guideline states that no more than the estimated number of required devices should be produced in the manufacturing process.

For the requirements (2)–(5) listed above, there is therefore no immediate need for pathology departments to act. However, pathology departments must address point (1), the “general safety and performance requirements”, which are described in Annex I of the IVDR.

## Implementation of annex I in pathology

Pathology departments in Germany are accredited as inspection bodies by the *Deutsche Akkreditierungsstelle GmbH* (DAkkS)—the national accreditation body of the Federal Republic of Germany—according to standard DIN EN ISO/IEC 17020. The accreditation focuses on the expert assessment of the pathologist and thus the confirmation of the professional competence of the inspection body [7]. The requirements of the standard EN ISO 15189 must also be considered [4]. The Institute of Pathology at the University Hospital Heidelberg (IPH) has been accredited since 2007. This provides an established, sound and independently audited quality management structure into which the requirements of the IVDR can be integrated. Article 5 (5) (c) of the IVDR requires a quality management system in accordance with the standard EN ISO 15189 but does not stipulate accreditation [8].

## Annex I: general safety and performance requirements

**Fig. 2:**
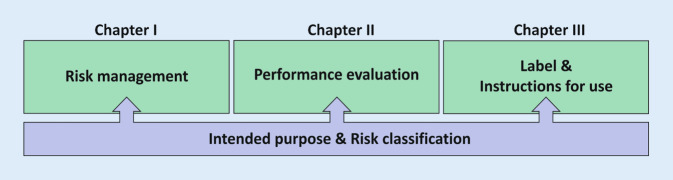
Annex I is subdivided into three chapters. Chapter I addresses the establishment of risk management. Chapter II contains the performance record as a central topic. Chapter III contains requirements for the labelling and instructions for use of IVDs. The basis of all chapters and requirements is the intended purpose and the associated risk classification. The intended purpose of the IH-IVD influences the effort required to implement the three central elements

All IVDs, whether commercial CE-IVDs or IH-IVDs manufactured in-house, must meet the general safety and performance requirements (Article 5 (5)). These requirements are intended to ensure and demonstrate that the devices are safe for patients and users, that potential risks are known and controlled, and that the devices are fit for their intended purpose. As a manufacturer and user of IH-IVDs, pathology departments must ensure and demonstrate compliance with Annex I. Annex I is divided into three chapters (Fig. 2):

- Chapter I: general requirements (→ focus on risk management)
- Chapter II: requirements regarding performance, design and manufacture (→ focus on performance evaluation)
- Chapter III: requirements regarding information supplied with the device (→ focus on labelling and instruction for use)

The intended purpose forms the basis for the requirements from Annex I. It thus determines the effort required for the requirements of Chapters I, II and III and the amount of the associated documentation.

### Intended purpose and risk classification

Defining the intended purpose enhances patient and user safety by making it less likely that the device will be used for purposes other than those intended and by preventing potential misuse. Furthermore, the “general safety and performance requirements” in Annex I are based on the intended purpose of the device. The intended purpose, therefore, has a decisive influence on the workload for the three central topics in Annex I (Fig. 2). “Intended purpose means the use for which a device is intended according to the data supplied by the manufacturer on the label, in the instructions for use […] or as specified by the manufacturer in the performance evaluation” (see Table 2 for a full definition).

The intended purpose is decisive for the classification of the device. The IVDR describes seven rules to classify IVDs, according to their intended purpose, into four classes A, B, C and D with increasing individual and public risk (Annex VIII). The more fatal the consequences of a possible misdiagnosis for the patient (e.g., in the case of life-threatening diseases) and the greater the risk to the public (e.g., in the case of transmissible agents of life-threatening diseases), the higher the risk class.

**Fig. 3:**
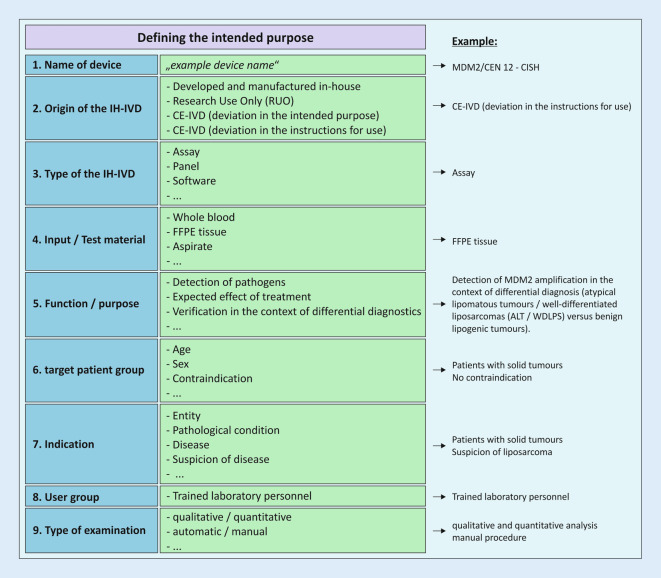
Formulation of the intended purpose for in-house in vitro diagnostic medical devices (*IH-IVD*). Several components (*blue*) are included and defined (*green*) as part of the determination of the intended purpose. The documentation is device specific, e.g., in the form of a checklist on a standard form

IPH has implemented a new standard operating procedure (SOP) in the existing QM documentation to ensure a uniform and comparable device-specific formulation of the intended purpose. The SOP describes how the intended purpose for IH-IVDs is formulated and how the risk classification is carried out. Several components (device name, type of IH-IVD, test material, function or purpose, indication, patient group and scope) are defined specifically for the device (Fig. 3). All these components are checked against a standard checklist to define the intended purpose. The same form is then used to classify and document the risk according to Annex VIII of the IVDR. Guidance document MDCG-2020-16 of the Medical Devices Coordination Group [12] also assists with classification.

## Annex I, Chapter I: general requirements

Chapter I focuses on device-specific risk management for the manufacture and use of IH-IVDs. IH-IVDs must be fit for purpose and safe; they must not compromise the safety of patients, users or third parties. Any risk associated with their manufacture and use must be acceptable in relation to the benefit to the patient and consistent with a high level of health protection and safety (IVDR, Annex I (1)). The IVDR defines risk as the combination of the probability of occurrence of harm and the severity of that harm (Table 2).

**Fig. 4:**
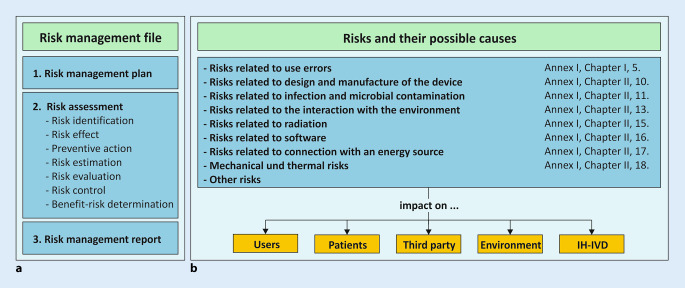
Device-specific risk management. **a** Risk management file: documentation of the risk management process. The risk management file represents the totality of the device-specific risk documentation. **b** Risk categories: Annex I describes several safety requirements that an in-house in vitro diagnostic medical device (IH-IVD) must meet. These requirements are considered device specific in the risk assessment and the possible effects (*yellow*) are analysed

For the implementation of device-specific risk management, a new SOP was created for the IPH QM documentation. This SOP describes creation of a device-specific risk management file (Fig. 4a). The risk management file contains the required risk management plan, the risk assessment with its subcomponents and a risk management report.

### Risk management plan

A risk management plan must be defined and documented for each IH-IVD. At IPH, we use a device-specific form to determine which “risk categories” are applicable to the IH-IVD under consideration (Fig. 4b). Risks that can be excluded by the intended purpose and manufacture are not considered further. The considered risk categories are based on the safety requirements described in Annex I, Chapter II. Thus, many of the safety aspects required by Annex I can be considered and included in the risk assessment.

**Fig. 5:**
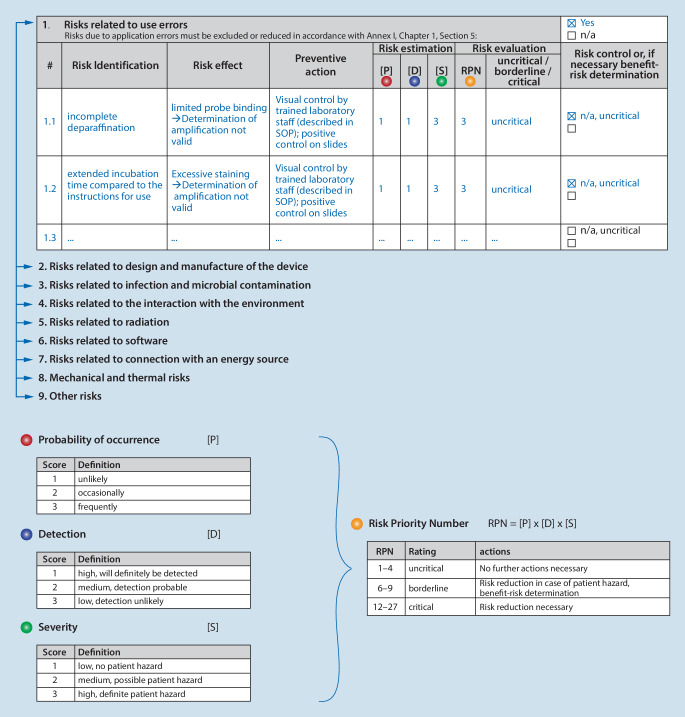
Risk assessment based on an example in the category “use errors”. All identified risks are analysed accordingly. This is done in tabular form for all risk categories shown in Fig. 4b

The responsibilities in the risk management process, the evaluation parameters for risk assessment and the criteria for accepting risks are defined in the device-specific risk management SOP. Risk assessment is performed on the basis of probability of occurrence [P], detection [D] and severity [S]. The risk scoring is based on the risk priority number [RPN]. This is determined by multiplying the risk assessment values (RPN = [P] × [D] × [S], Fig. 5). The higher the RPN, the higher the underlying risk potential. The resulting RPN values are used to determine control actions. The selection of the most appropriate risk reduction solutions shall be made in accordance with Annex I, Chapter I, 4:

1. the risk is eliminated or reduced as far as possible;
2. if the risk cannot be eliminated, appropriate protective measures are taken, including the establishment of warning mechanisms (e.g., controls);
3. safety information on residual risks (e.g., warnings and precautions, contraindications) is provided, e.g., in SOPs or protocols. These are specifically discussed in user training (e.g., as part of initial training).

### Risk assessment

The risk assessment is carried out by the personnel with the greatest expertise in the manufacture and use of the IH-IVD under consideration (e.g., laboratory personnel, scientist, pathologist, bioinformatician). Potential risks are considered for users (e.g., handling of hazardous substances), for patients (e.g., false positive/negative results), for potential third parties (e.g., exposure to hazardous substances), for the environment (e.g., disposal) and for the device itself (e.g., storage conditions, shelf life; Fig. 4b).

The risk assessment comprises the following components and is documented device specifically and in tabular form (Fig. 5):

- Risk identification: description of the risk and its root cause.
- Risk effect: description of the possible consequence or harm for users, for patients, for third parties, for the environment or for the device.
- Preventive actions: description of the preventive actions to minimize the risk.
- Risk estimation: based on the probability of occurrence, probability of detection and severity of harm.
- Risk evaluation: based on the risk priority number (RPN).
- Risk control/benefit–risk analysis: if necessary, description of further measures and control mechanisms (e.g., user training).

Any risk associated with manufacture and use must be acceptable in relation to the benefit to patients and compatible with a high level of health and safety protection (Annex I, Chapter I, 1.).

### Risk management report

The risk management report shall confirm that any residual risk associated with the manufacture and use is controlled, acceptable and consistent with a high level of health protection and safety in relation to the benefit to the patient. Both the residual risk associated with each hazard and the overall risk must be considered acceptable. All risk management documents are available to the user group through the QM system. In addition, they are informed separately about possible residual risks (e.g., within the specific SOP, in the context of documented training or protocolled meetings).

### Risk surveillance

Risk management must be a continuous iterative process that is regularly and systematically updated (Annex I, Chapter I, 3.). With the help of the failure analysis, correction and improvement processes established in the QM system (such as failure and action management, regular performance of internal and external audits and participation in round-robin tests), possible incidents related to IH-IVDs are recorded and reviewed. Ideally, if no incidents occur, the risk management file is reviewed at least every 2 years to ensure that it is up-to-date and valid. This review is also documented in the risk management file.

## Annex I, Chapter II: requirements regarding performance, design and manufacture

Chapter II describes requirements for performance, design and manufacture of IVDs. The safe design and manufacture of IH-IVDs have already been considered and assessed in the risk management file. According to the IVDR, the performance of a device is the ability “to achieve its intended purpose as claimed by the manufacturer” (note: for IH-IVDs → pathology departments; Table 2). Through the analysis and evaluation of defined performance characteristics, the scientific validity, analytical performance and, if applicable, clinical performance are determined and verified before the devices are used (= performance evaluation; Table 2). Examples of the performance characteristics to be considered are listed in Chapter II, 9.1. and are determined on the basis of the device-specific intended purpose defined “by the manufacturer” (see below and Fig. 3). The intended purpose thus significantly influences the scope of the validation and decides which of the performance characteristics listed in Chapter II, 9.1., must be demonstrated and which can be excluded. A publication of the IVDR subgroup of the German *Arbeitsgemeinschaft der Wissenschaftlichen Medizinischen Fachgesellschaften e.* *V.* (AWMF; Association of the Scientific Medical Societies in Germany) provides guidance on the performance evaluation of in-house methods for the detection of infectious pathogens [14].

**Fig. 6:**
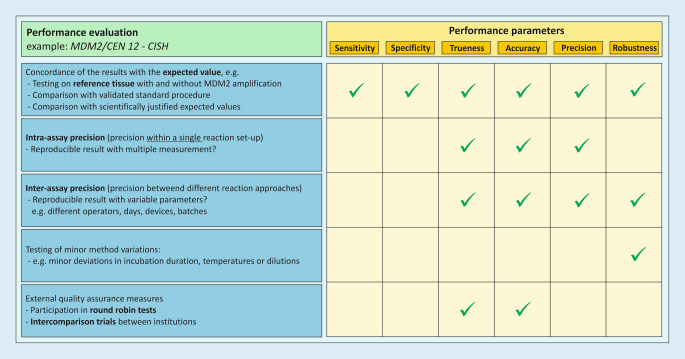
Definition of the performance characteristics to be determined using the example “MDM2/CEN 12—CISH”. Based on the intended purpose (Fig. 3), the scope of the validation is defined. It is checked which performance characteristics are applicable. Other performance characteristics listed as examples in Annex I, Chapter II, 9.1, are not applicable to this example

Method validation at IPH is based on guidelines of the Pathology/Neuropathology Sector Committee, which is responsible for the interpretation of the accreditation requirements according to DIN EN ISO/IEC 17020 in the field of pathology [2, 3]:

1. Definition of the performance characteristics to be determined (example Fig. 6):
  Based on the intended purpose, the scope of the validation is determined. It is checked which performance characteristics are applicable. The defined performance characteristics are documented in a validation-specific form.
2. Description of the test procedure:
  The results and findings from the development and establishment phase are included in the specific SOP. The validation is performed according to this SOP.
3. Determination of performance characteristics:
  Proof that the specified quality requirements are met in the specific case.

The concluding performance evaluation is made in the device-specific validation report. This requires qualified personnel to decide whether the quality requirements for the method have been met and whether it can be used for the intended investigations in accordance with the intended purpose. Ongoing verification of the validated procedure is ensured by appropriate control mechanisms during operation. This ensures that the procedure is reproducibly stable and robust. The established QM system with its correction and improvement processes described above also applies here.

## Annex I, Chapter III: requirements regarding information supplied with the device

Chapter III specifies requirements for the information supplied with the device. “Each device shall be accompanied by the information needed to identify the device and its manufacturer, and by any safety and performance information relevant to the user or any other person, as appropriate” (Annex I, Chapter III, 20.1.). This is done through labelling and instructions for use, for which Chapter III provides specifications. Instructions for use are defined as follows: “‘instructions for use’ means the information provided by the manufacturer to inform the user of a device’s intended purpose and proper use and of any precautions to be taken” (Table 2).

In accordance with the requirements for IH-IVDs set out in Article 5 (5), pathology departments are both manufacturer and user, as the devices may not be transferred to another legal entity. Thus, all relevant information on the manufacture, safety and performance of the IH-IVD is available to the user group at all times. Within the QM system, the intended purpose, risk assessment and performance evaluation/validation documents are available to the user group at all times. In addition, there are device-/process-specific SOPs in the QM documentation. These are either prepared by the user group itself or the users are trained on them as part of the training program. These SOPs cover important aspects of the safe use of in-house methods and, like risk management, are regularly and systematically updated. The safe use of IH-IVDs in the laboratory, which is necessary to meet safety requirements, also includes relevant information on substances or mixtures classified as hazardous, e.g., in the form of safety datasheets and laboratory safety instructions.

## Discussion and conclusion

Commercial CE-IVDs and IH-IVDs are specifically used in pathology for optimal diagnosis and individualized therapy [8]. With Recital 29 and its concretization in Article 5 of the IVDR, the legislator recognizes the benefit and necessity of in-house developed tests and allows their use for optimal patient care under certain conditions [8, 17]. As manufacturers and at the same time users of IH-IVDs, health institutions like pathology departments must ensure compliance with Article 5 (5) of the IVDR and fulfil the safety and performance requirements listed in Annex I. Then, all other requirements of the IVDR do not apply to IH-IVDs.

In January 2023, the Medical Device Coordination Group (MDCG) document MDCG-2023‑1 was published for health institutions, providing guidance on some of the requirements of Article 5 (5) [13]. The MDCG and its tasks are described in Articles 98 and 99 of the IVDR. According to Article 99 (c), it has the task of developing guidelines for an effective and harmonized implementation of the IVDR, but these are not legally binding.

Many professional societies and associations are committed to the practical implementation and content shaping of definitional gaps and vagueness in the requirements, and new networks have formed to use their academic knowledge and expertise to provide assistance. They provide support, for example, through guidelines and templates, as well as through discussion events, workshops and the exchange of experiences. The IVDR subgroup of the AWMF provides numerous templates and supporting documents in German and English (e.g., on risk management and performance evaluation) on its website [1]. The recommendations of the *Bundesverband Deutscher Pathologen e.* *V. *(BDP; Federal Association of German Pathologists) [9–11], which describe the implementation of IVDR in pathology during the implementation phase, are also helpful.

Such information sources are an important step for institutions to implement and harmonize compliance with the requirements of Article 5(5). Academic networking structures are also important for interpretation of the IVDR and its practical implementation.

**Fig. 7:**
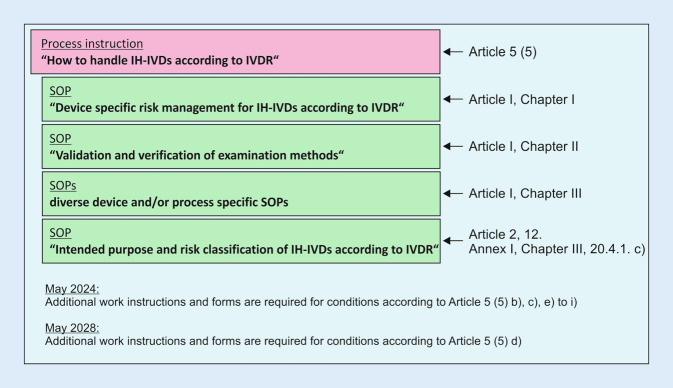
Implementation of the Annex I requirements for in-house in vitro diagnostic medical devices (*IH-IVD*) in the established quality management system of the Institute of Pathology at Heidelberg University Hospital (IPH)

In Germany, around 100 institutes for pathology or neuropathology are accredited according to DIN EN ISO/IEC 17020 [7]. The implementation of the requirements of the IVDR for IH-IVD thus takes place within a solid, established and independently audited quality management structure, in which many requirements have already been implemented (e.g., continuous verification of methods by quality controls, user training or failure management). To comply with Annex I of the IVDR, the Institute of Pathology in Heidelberg (IPH) has created several new operating procedures and integrated them into the existing DIN EN ISO/IEC 17020-compliant QM documentation (Fig. 7). In some cases, existing QM documents were used, but these had to be adapted to the requirements of Annex I.

The intensive internal examination of the requirements of the IVDR (e.g., through gap analysis), the preparation of the QM documents listed in Fig. 7 (SOPs and forms), their implementation in the existing QM system, the necessary training and, above all, the device-specific documentation provided for in the IVDR through the expertise of the specialist staff are very resource intensive. For the device-specific documentation, the IH-IVD must first be defined and the area of application specified. Depending on the strategy, this can be done for an entire process chain, or modularly for individual links or elements, and takes the form of a documented intended purpose. Based on this determination, the device-specific risk management, which goes beyond general risk management within the scope of accreditation, performance evaluation and instructions for use (as SOPs) are established in accordance with the QM manual. A new challenge is posed by in-house software solutions, which now also fall under the definition of an IVD according to the IVDR.

For a university institution like IPH, ensuring compliance with the IVDR—in addition to the core tasks of patient care, teaching and research, the further development of methods for optimal and targeted personalized diagnostics and maintenance of the constantly evolving QM system—represents an additional major challenge in terms of personnel and time.

## Conclusion for practice

- Since May 2022, health institutions have to comply with Annex I of the IVDR for IH-IVD.
- Accreditation according to DIN EN ISO/IEC 17020 is not required, but it provides a solid basis for compliance with Article 5 (5) of the IVDR.
- Carry out gap analysis → Article 5 (5) vs. established quality management system → implement IVDR requirements that are not yet mapped.
- Existing established quality management structures can and should be used and extended if necessary.
- Guidelines, templates and checklists will assist in the implementation of the requirements for IH-IVD.
- With the aim of harmonizing implementation, various academic networks, associations and professional societies are working on the development and publication of supporting guidance documents (e.g., AWMF, BDP).

## Declarations

For this article no studies with human participants or animals were performed by any of the authors. All studies mentioned were in accordance with the ethical standards indicated in each case.

The supplement containing this article is not sponsored by industry.
